# Optimizing nutrition, diet, and lifestyle communication in GLP-1 medication therapy for weight management: A qualitative research study with registered dietitians

**DOI:** 10.1016/j.obpill.2024.100143

**Published:** 2024-10-13

**Authors:** David Despain, Brenda L. Hoffman

**Affiliations:** School of Communication and Journalism, Stony Brook University, Stony Brook, NY, USA

## Abstract

**Background:**

This qualitative study used semi-structured interviews to examine registered dietitians’ perspectives on diet, nutrition, and lifestyle communication for patients on GLP-1 medications for obesity management.

**Methods:**

Through in-depth interviews with registered dietitians, this research identifies elements that could improve both the efficacy of GLP-1 medication therapies and patient adherence.

**Results:**

indicate that a comprehensive approach, integrating patient communication with proactive management of side effects and ongoing lifestyle counseling, is essential for optimizing treatment outcomes.

**Conclusion:**

Key findings include the importance of using visual and metaphorical aids to improve understanding, the necessity for structured lifestyle programs, and the pivotal role of personalized diet plans. These insights offer valuable directions for enhancing patient care and formulating clinical practices around the use of GLP-1 receptor agonist medications.

## Highlights

• This study interviewed dietitians on their views regarding diet and lifestyle education for patients on GLP-1 medications.
• Participants emphasized the need for better communication and managing expectations during obesity treatment.
• Managing appetite suppression and side effects with dietary advice promotes adherence and prevents nutritional deficiencies.
• Tailored nutritional plans based on patient needs can aid in long-term weight maintenance and better health outcomes.
• Addressing mental health and body image with psychological support builds sustainable habits alongside GLP-1 medication use.

## Graphical abstract

**Figure:**
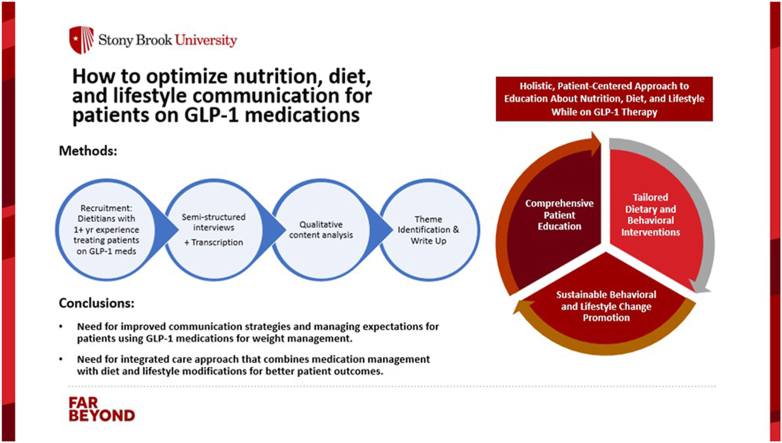

## 1 Introduction: GLP-1 receptor agonists

Glucagon-like peptide-1 receptor agonists (GLP-1 RAs) represent a major advancement in the pharmacological treatment of type 2 diabetes and obesity [1]. They operate by enhancing insulin secretion in response to food intake without risking hypoglycemia and effectively reduce appetite and body weight [[2], [3], [4], [5]]. However, the successful implementation of GLP-1 RA therapy is not without challenges; these include gastrointestinal adverse events (GI AEs), potential risks of undernutrition, loss of lean body mass, and the propensity for weight regain after treatment cessation [[6], [7], [8], [9]]. This study aims to investigate the roles that healthcare professionals, particularly dietitians, play in optimizing the therapeutic outcomes of GLP-1 RAs through diet and lifestyle education. By focusing on the communication and behavioral strategies that enhance patient engagement and adherence, this research seeks to develop an understanding of the comprehensive approach required to effectively manage these medications.

## 2 Methods

### 2.1 Research design

This study employed a qualitative research design, using semi-structured interviews to explore the perspectives of registered dietitians on nutrition, diet, and lifestyle education for patients on GLP-1 RAs for obesity. The qualitative content analysis framework was applied following the procedures and measures outlined by Graneheim and Lundman (2004) to ensure rigor and depth in analyzing the interview data [10].

### 2.2 Participant selection

The following criteria were used to determine if healthcare providers should be involved in the study: (1) at least 18 years of age; (2) must be one of the following: physicians, nurse practitioners, physician assistants, registered dietitian nutritionists, or other relevant healthcare professionals; (3) have one or more years of experience being involved in the care of patients on GLP-1 RAs for weight management; (4) must be currently involved in the care of patients on GLP-1 RAs for weight management, as shown in Table 1. Additionally, healthcare providers were excluded if they indicated financial or professional conflicts of interest related to GLP-1 medications, such as receiving compensation from pharmaceutical companies or having personal investment in related products. The recruitment of participants occurred via email and direct messages over social media platforms. All recipients were also asked to share the invitation with colleagues who may be interested in participating in the study. Once recruited to participate in the study, the healthcare providers received an informed consent document.

**Table 1:** Participant inclusion criteria checklist.

At least 18 years of age
Must be one of the following: physicians, nurse practitioners, physician assistants, dietitians, and other relevant healthcare professionals
Must have 1+ years of experience treating patients with GLP-1 medications for weight loss
Must be currently treating patients with GLP-1 medications for weight loss

Thirteen registered dietitians, experienced in managing patients on GLP-1 RAs, were recruited via email for this study. Recruitment attempts incorporated a wide variety of healthcare providers with experience in managing patients on GLP-1 medications, including physicians, endocrinologists, nurse practitioners, and other relevant healthcare professionals. However, responses of interest that were received were primarily from registered dietitians, or registered dietitian nutritionists, who eventually made up the entirety of the study participants. These registered dietitians were recruited through the Academy of Nutrition and Dietetics EatRight.org webpage by clicking on “Find a Nutrition Expert”. Despite attempts to include a broader range of healthcare providers, recruitment was not successful beyond registered dietitians.

### 2.3 Data collection

Data were collected through semi-structured interviews, each lasting approximately 30–45 minutes. The interviews were conducted via the secure Zoom video conferencing platform, as provided by Stony Brook University. An interview guide was developed to ensure consistency in the topics covered, while allowing flexibility for participants to introduce new ideas and share personal experiences. The interviews focused on exploring communication strategies, challenges faced in patient education, and perceptions of the effectiveness of GLP-1 medications. The semi-structured interview protocol included open-ended questions, as shown in Table 2, that facilitated an in-depth discussion of various themes.

**Table 2:** Questionnaire used as part of the Interview Guide.

**1.** Nutrition Education Practices How do you approach discussion about nutrition, diet, and lifestyle with patients while they're undergoing GLP-1 treatment for weight loss? What kinds of nutrition education materials or resources or resources are typically provided to patients undergoing GLP-1 treatment? (Brochures, videos, support groups, etc.)
**2.** Challenges and barriers In your experience, what are some of the challenges or barriers encountered in providing nutrition education to patients receiving GLP-1 treatment? How would you describe the effectiveness of nutrition education efforts on adherence and patient outcomes?
**3.** Strategies for improvement What potential strategies could enhance nutrition education and support for patients on GLP-1 medications? What strategies would you recommend that have been successful in promoting dietary adherence, managing adverse events, or encouraging lifestyle changes?
**4.** Patient engagement Would you reflect on any experiences or examples regarding diet or lifestyle education that has empowered GLP-1 patients to make sustainable lifestyle changes? Would you have any additional comments about strategies to address concerns or misconceptions related to nutrition or dietary recommendations while on GLP-1 medications?

### 2.4 Data handling and analysis

All interviews were taken from the researcher's home and recorded using the Zoom application as provided by Stony Brook University. These were audio-recorded with consent from the participants and transcribed verbatim to ensure accuracy in data representation. Participant names and identifying information were stored separately from the informed consent and interview scheduling documents. Data collected during interview sessions were only recorded as Participant X, etc. Numbers were randomly assigned to participants so that identifying information could not be traced back to individual responses. Transcripts were anonymized by removing names and any identifying information to maintain confidentiality. At the start of the interviews, participants were informed of this information and that they can opt out of any question or the interview at any point after the interview had started.

Qualitative content analysis was performed on the transcripts to identify, analyze, and report patterns (subthemes) within the data. Following guidance from the literature on the process of qualitative content analysis [10,11], the analysis process involved several steps: (1) each interview transcript was treated as a single unit of analysis; (2) “meaning units” referred to relevant quotes that contained key information related to the study objectives were identified; (3) “condensed meaning units” were the meaning units condensed into a shorter form while preserving their core content; as shown in Table 3, “categories” and “subthemes” were identified based on their content and context similarities. Subsequently, an “overarching theme” was developed by interpreting the underlying meaning across categories.

**Table 3:** Examples of how quotes were used in the process of analysis of categories and subthemes.

Meaning Unit	Condensed Meaning Unit	Category	Subtheme
“I liken this to when we first started having bariatric surgeries … Incredibly poor education."	Comparison of current practices to past poor educational strategies	Quality of education	Patient Education and Management
“If you're eating once a day, it's very difficult to get adequate vitamins and minerals in the ratio of macronutrients that you need."	Challenges of limited eating frequency on nutrient intake	Nutritional challenges	Medication & Side Effect Management
“I have to explain how this happens … This is what's going on in your body."	Explanation of physiological response to medication	Patient understanding and expectations	Patient Education and Engagement
“Setting honest expectations about the side effects of medications … Is crucial."	Importance of managing expectations regarding side effects	Patient understanding and expectations	Patient Engagement and Education
“Physicians should always refer to a dietitian when prescribing these medications."	Importance of dietitian referral	Tailored diet recommendations	Patient Education and Engagement

### 2.5 Ethical considerations

Ethical approval for this study was granted by the Institutional Review Board (IRB) at Stony Brook University. All participants provided informed consent, which was structured according to a template approved by the IRB. Participants were fully informed of their rights, including the ability to withdraw from the study at any time, and the confidentiality of their responses was assured. No participants chose to withdraw, and all scheduled interviews were completed in full. All participant data were securely stored on a research server at Stony Brook University, accessible only to the study's author and the principal investigator. This secure storage ensured the protection of sensitive information and compliance with ethical standards for handling research data. Participation was entirely voluntary, and informed consent was verbally confirmed by each participant during their interview.

## 3 Results

### 3.1 Overarching theme: Need for optimizing nutrition, diet, and lifestyle communication in GLP-1 medication therapy

The overarching theme identified characterizes the overall nature of the subthemes and categories reflected from the discussions with dietitians: “Need for optimizing nutrition, diet, and lifestyle communication in GLP-1 medication therapy.” There were three subthemes identified as part of the overarching theme, which directly linked to research questions. These are presented consecutively in the following paragraphs.

### 3.2 Subtheme 1: patient understanding and engagement

The first subtheme addresses existing deficiencies in education relating to nutrition, diet, and lifestyle for patients on GLP-1 therapies. The deficiencies include poor management of expectations, subpar quality of educational materials relating to GLP-1 medication, and concerns over lack of personalized dietary guidance. Addressing these issues is needed to ensure that patients are well-informed and actively involved in their treatment to improve outcomes on GLP-1 RAs for managing obesity and type 2 diabetes.

*Patient understanding and expectations*. This category comprised setting realistic expectations and understanding about GLP-1 RAs among patients. For instance, one participant said, “Setting honest expectations about the side effects of medications … is crucial. It's a lot easier to manage when my patients know what to expect.” The participants highlighted the necessity of dispelling myths about GLP-1 RAs being a “magic pill,” emphasizing that these are not cure-alls and require active participation and complementary lifestyle changes. For example, one dietitian stated, “In the initial discussion if they're on it or if they're considering it is always that you know it's not a panacea; it's not going to fix everything.” Additionally, several participants shared that they often addressed the history and differences between available GLP-1 medications. For instance, one dietitian shared, “I know patients tell me that it’s new. It was never or it's not FDA approved for obesity. That's a big one. So, I will explain to them that there's no difference between Wegovy and Ozempic … they are the same thing. I tell them it's the difference between like a tall and a grande coffee … it's the same contents. It's just a different amount.”

*Quality of education.* Dietitians emphasized the role of quality education in enhancing patient engagement and managing GLP-1 RA therapy. The need for comprehensive educational strategies was mentioned frequently, with dietitians advocating for better resources that address the specific needs of patients. The critique of current educational practices included calls for more effective and clear communication strategies. One dietitian stated, “We're missing something like we had released this medication, but with little education and not as big of involvement with dietitians, who are experts in the field.” Several of the participants drew historical parallels to previous weight loss therapies, including one participant who stated, “I liken this to when we first started having bariatric surgeries … incredibly poor education.” However, some participants noted the effectiveness of using varied educational tools and adapting materials to improve understanding: “I think people are definitely visual learners. I do use different handouts and things while I'm talking to people; the two that I use the most commonly are the ‘Healthy Eating Plate’ and ‘MyPlate’ models.” The Healthy Eating Plate refers to the visual guide created by nutritionists at Harvard T.H. Chan School of Public Health and MyPlate is a visual guide developed by the U.S. Department of Agriculture. Another visual guide mentioned by dietitians was the “The Athlete's Plate,” a visual tool designed for sports dietitians working with athletes, developed by the University of Colorado-Colorado Springs. These visual tools are helpful for patients to more easily comprehend portion sizes while being able to obtain sufficient nutrients such as protein and dietary fiber.

*Tailoring diet recommendations.* Personalization of dietary recommendations to align with individual lifestyles was a central theme in the dietitians' approach to patient communication. The participants discussed the importance of understanding patient lifestyles, preferences, and specific health needs to provide effective dietary guidance. For example, one dietitian described their method: “I spend the good majority of my sessions really asking a lot of questions just to understand lifestyle, sleep habits, schedule for eating … because for me that really helps tailor the recommendations to be very specific.” Many of the participants also advocated for the dietitian's role in supporting therapy, such as with crafting personalized diet plans but also enhancing patient adherence and satisfaction with the treatment process.

Through these insights, it's suggested that effective patient communication and engagement in GLP-1 therapy hinge on setting accurate expectations, delivering high-quality education, and personalizing dietary recommendations to meet individual needs. These elements are important for improving treatment outcomes and ensuring that patients are well-informed and actively involved in their own care.

### 3.3 Subtheme 2: medication and side effect management

The second subtheme addresses health communication strategies that are most effective in enhancing patient compliance and mitigating side effects while on GLP-1 medication. The management of side effects and nutritional challenges with GLP-1 RAs requires a nuanced understanding of both the pharmacological effects of the drugs and the dietary needs of patients. It involves a balance of medical management, dietary adjustments, and continuous patient communication to ensure that the benefits of the medications are maximized while minimizing adverse effects and nutritional risks.

*Nutritional challenges.* Many participants discussed the challenges associated with ensuring adequate protein and overall nutrient intake when appetite is suppressed by GLP-1 medications. For instance, one dietitian noted, “The biggest challenge in my opinion on these meds … the first two months or six weeks at least … appetite is just gone.” Another participant who highlighted protein due to its muscle-maintenance role stated, “We focus a lot on adequate protein … you want to get protein at every eating occasion.” The risk of malnutrition, especially when patients experience rapid weight loss, was another concern. As one dietitian expressed, “Early satiety … interferes with oral intake. I feel like there's a lot of undiagnosed malnutrition.”

*Side effect support.* The participants reported that GI AEs affect patient compliance and quality of life. They shared various approaches to mitigate these issues, such as advising smaller, more frequent meals and incorporating more liquids to ease nausea. For example, one practitioner explained, “We can also really target some of their side effects … Simple things like ginger tea, peppermint tea, drinking enough water … helps them manage their symptoms.” The majority of GI AEs were said to center around nausea and diarrhea, as well as eructation (“sulfur burps”); however, constipation has also been a problem leading to recommendations of incorporating more dietary fiber, hydration, and physical activity. A dietitian highlighted the importance of customization of diet management depending on the adverse events experienced, “Everybody's different. Some people can handle a little bit more fat, some people can handle a little bit more fiber, some people can't … so we need to kind of do an elimination diet approach of writing everything down and saying, ‘Okay, what could have caused the problem?”

*Medication management.* In managing the use of GLP-1 RAs, the participants emphasized the importance of a comprehensive approach that includes patient communication on the pharmacodynamics of the drugs, setting realistic expectations around weight loss, and proactive management of side effects. A participant stated, “I think patients tend to downplay their symptoms with the medications because they know it's working; they're losing weight, you know, so they'll stay on a medication, even if they are very uncomfortable.” Discussions often revolve around educating patients on how the medications work to reduce appetite and slow gastric emptying, thereby aiding in weight management but also presenting challenges in nutritional intake. There was also a focus on adjusting medication dosages to alleviate side effects, with strategies tailored to individual patient tolerances and lifestyle considerations. One dietitian shared her approach to help patients manage side effects and feel more in control of their own treatment: “Another thing I tell my patients is that, yeah, there's a most rapid way to uptitrate your dose, but you don't get extra credit for doing it fast. You could do it a little slower.”

### 3.4 Subtheme 3: Behavioral and lifestyle modification

This third subtheme addresses challenges that healthcare providers face when educating on diet and lifestyle modifications for those on GLP-1 therapy. These insights from dietitians describe the need for a multifaceted approach based on patient education and motivation for improved care with GLP-1 therapies for weight management. The emphasis on encouraging behavior change, providing comprehensive care, including post-treatment, and ensuring long-term sustainability highlights the complexity of treating conditions like obesity and type 2 diabetes effectively.

*Encouraging behavior change.* There's a need for strategies to encourage lifestyle changes among patients using GLP-1 RAs. The participants emphasized the need for patients to actively engage in modifying their behavior to ensure successful outcomes. For instance, one participant stressed the importance of integrating exercise with medication: “I get them to make that commitment that okay you have to agree to increasing your activity or exercise.” Another highlighted the need for dietary changes, stating, “And also get them to agree they have to change their eating habits; they can't just eat 500 calories of Twinkies you know and consider themselves healthy.” There was a consistent pattern among the dietitians' views that GLP-1 RAs should be viewed as tools that support, but do not replace, the necessary lifestyle adjustments needed for long-term health improvements.

*Comprehensive care.* A holistic approach is needed in managing patients on GLP-1 RA therapy with the importance of considering both physical and mental health aspects. Participants pointed out the need for a broader focus beyond simple medication management. For example, one dietitian noted, “I'm looking not only at my patient's physical health but also their mental health,” indicating the dual focus on comprehensive care and assistance with possible internalized weight stigma. One dietitian stated, “The stigma happens from social media exposure, TV … I do try to explain that there's no one size fits all … I build more of something called body positivity where I send them daily reminders that your body doesn't define who you are and you're beautiful as you are.” Additionally, the inclusion of behavioral counseling as a component was emphasized, with suggestions for incorporating techniques like Dialectical Behavioral Therapy (DBT) for patients with impulse control issues: “Like for binge eating disorder, they need to be in a DBT course.” One participant emphasized the role of dietitians by stating, “If a physician is prescribing one of these medications, they should always be referring to a dietitian because they don't have the time to have this discussion with a patient.” This viewpoint was shared by other dietitians describing the necessity of involving dietitians in the treatment process, not only to provide detailed nutritional counseling tailored to the patient's needs but also to handle the complexities of diet and weight management that go beyond the physician's general scope.

*Long-term sustainability***.** There is a need for patients to recognize the importance of sustainable health habits that extend beyond the period of medication use. Participants discussed strategies to ensure that behavior changes are maintained in the long term, emphasizing education and gradual integration of healthier habits. One dietitian shared, “Rather than focusing on a medication or a disease state right away, I like to kind of take a step back and just talk about lifestyle and then kind of intertwine the recommendations to the lifestyle. I have found that just works really well regardless of the patient.” This approach aims to weave medication into a broader lifestyle modification plan, ensuring that patients develop habits that can sustain health benefits even after the cessation of medication. As one participant put it, “It's a teaching tool, just like training wheels on a bicycle … the [patient] needs to learn what it feels like to ride a bike first. And then, you start taking them off.” Additionally, there exists a need for counseling patients who express serious concerns about having to come off medication. For example, a participant stated, “I had one client that I saw for over a year … she was doing really well … but she was terrified of getting off of the medication, because she was so worried about gaining weight back.”

## 4 Discussion

### 4.1 Research insights

The findings from this small study provide valuable insights into the roles of patient communication, medication management, and behavioral modifications in the context of GLP-1 RA therapy for weight management. Integrating qualitative data from registered dietitians promotes greater awareness and understanding of the importance of a comprehensive approach required to optimize the outcomes of GLP-1 RA therapy. The importance of patient understanding and setting realistic expectations was a recurring theme from the qualitative analysis. The participants emphasized the necessity of clarifying that GLP-1 RAs are not a standalone solution but a tool that enhances the effectiveness of lifestyle modifications. The management of side effects and nutritional challenges associated with GLP-1 RAs requires careful consideration. Concerns over undernutrition, particularly loss of lean body mass, due to low protein intake is consistent with previous publications [7,12,13]. Additionally, dietitian concerns related to decreased fiber intake and dehydration as a contributor to GI AEs is consistent with the scientific literature [6,9]. The findings also indicate that improved communication strategies, particularly through patient interaction with a dietitian, could have an important role in supporting patients through these challenges by providing tailored advice on diet modifications and side effect management. Moreover, the discussion highlights the need for ongoing adjustments to medication dosages and dietary recommendations to improve patient comfort and compliance, as has been used in studies previously [[14], [15], [16]]. In addition, sustainable behavior change was highlighted as essential for the long-term success of GLP-1 RA therapy. This is consistent with previous studies demonstrating that lifestyle changes, such as continuing an exercise program, is effective in assisting with weight loss maintenance [17,18].

Participants in the study also advocated for an integrated approach that addresses both physical and mental health aspects of obesity management. The role of dietitians was particularly emphasized, with providers advocating for their involvement in comprehensive care. The problem of weight stigma, including internalized weight stigma, interfering with proper health care and behavior change as highlighted by various participants in this study has also been extensively discussed previously in the scientific literature [[19], [20], [21], [22]]. In this study, dietitians considered their roles to be pivotal in bridging the gap between medical treatment and behavioral health, providing essential dietary counseling, screening, and motivational support that complements the pharmacological aspects of GLP-1 RA therapy.

The need for comprehensive care emerged strongly from the qualitative content analysis, suggesting that there ought to be an integration of behavioral counseling with nutritional and medical management. While the study findings are based solely on the views of dietitians, these findings are consistent with other viewpoints of an integrative approach to obesity treatment that includes GLP-1 RA therapy combined with lifestyle counseling, including nutrition and culinary education and behavioral support [6,23]. The collaborative approach between physicians and dietitians is important for delivering holistic care that addresses all facets of patient health. This is consistent with previously published literature with viewpoints that dietitians bring value to patient-centered healthcare models [[24], [25], [26]]. For example, dietitians can assist with screening for eating disorders or other potential mental health conditions that could help with determining whether GLP-1 RAs are appropriate for use in therapy. This collaboration ensures that patients receive not only medical intervention but also the behavioral support necessary to make lasting changes. However, a previous systematic review of studies involving dietetic consultations found that effective communication skills are essential for dietitians for implementing proper patient-centered care to meet the needs of their patients with time constraints emerging as a particular barrier [26].

### 4.2 Implications for clinical practice

These findings suggest several implications for clinical practice. Firstly, there is a need for structured education programs that accurately inform patients about the benefits and limitations of GLP-1 RAs in the context of a comprehensive treatment plan. Secondly, healthcare providers should adopt an integrated care model that includes dietitians and mental health professionals to address the complex needs of patients undergoing GLP-1 RA therapy. The integration of detailed patient communication, proactive side effect management, and comprehensive lifestyle counseling can help to improve patient outcomes and adherence to GLP-1 RA therapy.

The development of strategies to promote long-term sustainability of lifestyle changes is critical for maintaining the benefits of treatment after the cessation of medication. In response to the need for improved patient communication on GLP-1 RAs, the qualitative analysis identified several strategic approaches aimed at enhancing the educational practices surrounding the use of these treatments. These strategies, as shown in Table 4, can be employed to assist in ensuring that patients fully understand and effectively manage their therapy to achieve optimal outcomes.

**Table 4:** Strategies for enhancing communication practices for GLP-1 medication guidance.

Subtheme	Strategy	Description
Patient Education and Engagement	Use of MyPlate or Similar Visuals	Use MyPlate or similar visuals to simplify meal planning and portion control, making it easy for patients to understand and apply nutritional guidelines daily.
	Metaphor Use in Communication	Employ metaphors, such as referring to GLP-1 medications as “training wheels,” to help patients understand that the medication is a temporary aid designed to teach and reinforce healthier habits, not a permanent solution.
Behavioral and Lifestyle Modifications	Structured Lifestyle Modification Programs	Develop structured programs that include gradual increases in physical activity and modifications in eating habits, ensuring that lifestyle changes complement medication use.
	Comprehensive Behavior Change Workshops	Organize workshops focusing on comprehensive behavior change, incorporating strategies to cope with medication side effects, enhancing self-management skills, and sustaining long-term health improvements.
Dietary Management and Nutritional Counseling	Interactive Dietary Counseling	Dietary counseling guides patients on how to manage their diet while on GLP-1 medications, focusing on overcoming challenges like reduced appetite and nausea.
	Tailored Nutritional Plans	Offer personalized nutritional planning that considers individual dietary needs, preferences, and lifestyle, with regular adjustments based on patient feedback and health progress.
Medication Management and Side Effects	Side Effect Management Guides	Create detailed guides that explain common side effects and provide practical tips for managing them, such as dietary adjustments and symptom relief techniques.
	Regular Monitoring and Feedback Sessions	Set up regular sessions to monitor patient responses to the medication, discuss their experiences, and adjust treatment plans as necessary to manage side effects effectively.

## 5 Limitation

While this study provides foundational insights into the effectiveness of various communication and management strategies for GLP-1 RA therapy, several limitations must be acknowledged. The qualitative nature of this study, relying on a small sample of interviews from registered dietitians, may introduce bias and limit the generalizability of the findings. Additionally, the perspectives of patients themselves were not directly included, which might affect the comprehensiveness of the insights regarding patient experiences and satisfaction. Future research should aim to incorporate a broader demographic, including other healthcare providers and direct feedback from patients to understand their perspectives and experiences fully. Quantitative studies could also be employed to measure the effectiveness of the identified communication and behavioral strategies in improving patient outcomes. Further exploration into the long-term impacts of these interventions, including differences between medications, on patient adherence and health outcomes would provide deeper insights into the chronic management of obesity and type 2 diabetes with GLP-1 RAs. Additionally, comparative studies between different educational materials and communication strategies could delineate more effective methods for enhancing patient understanding and engagement in their treatment plans.

### 5.1 Limitation regarding hydration

Additionally, this study's focus on dietary recommendations may not fully address broader clinical concerns, such as the important role of adequate hydration in maintaining renal function and preventing dehydration. The lack of a detailed discussion on hydration, fundamental for the safety and efficacy of GLP-1 RA treatment, is a notable limitation considering existing research that the medications can suppress water intake [27]. Future research should explore the importance of hydration in GLP-1 RA therapy to promote a comprehensive approach to patient care and education.

## 6 Conclusion

This study illuminates the critical role that enhanced communication practices and comprehensive care strategies play in the management of GLP-1 RAs for weight management.

• The insights gained advocate for a multidisciplinary approach that not only focuses on medication management but also emphasizes the importance of dietary guidance, behavior modification, and regular patient monitoring to optimize treatment outcomes.
• The integration of structured educational approaches, such as the use of visual aids and metaphors, alongside tailored lifestyle modification programs, provides a robust framework for supporting patients in their treatment journeys.
• Through the implementation of interactive workshops and personalized diet plans, healthcare providers can address the unique challenges posed by GLP-1 RA therapy, such as GI AEs and risk of undernutrition, improving patient engagement and adherence.

Moving forward, it is important to consider implementing these comprehensive strategies and refining them to meet the continuously evolving needs of the patient population, ultimately leading to improved health outcomes and quality of life for individuals undergoing GLP-1 RA therapy.

## Author contribution

The concept of the submission was by DD and BH. DD participated as the sole investigator and data collector. DD wrote the first draft. BH reviewed, edited, and approved the final submission and publication.

## Disclosures

DD is an employee of Nestlé Health Science located at 1007 US Highway 202/206, Bridgewater, NJ, 08807.

## Ethical adherence and ethical review

Ethical approval for this study was granted by the Institutional Review Board (IRB) at Stony Brook University. All participants provided informed consent, which was structured according to a template approved by the IRB.

## Declaration of artificial intelligence and AI-assisted technologies

During the preparation of this work, the authors did not use AI.

## Source of funding

No financial support was given for this qualitative research. While DD is an employee of Nestlé Health Science, no funding was provided for this research from the company.

## References

[1] Jastreboff A.M., Kushner R.F. (Jan 27 2023). New frontiers in obesity treatment: GLP-1 and nascent nutrient-stimulated hormone-based therapeutics. Annu Rev Med.

[2] Jastreboff A.M., Aronne L.J., Ahmad N.N. (Jul 21 2022). Tirzepatide once weekly for the treatment of obesity. N Engl J Med.

[3] Wilding J.P.H., Batterham R.L., Calanna S. (Mar 18 2021). Once-Weekly semaglutide in adults with overweight or obesity. N Engl J Med.

[4] Muller T.D., Finan B., Bloom S.R. (Dec 2019). Glucagon-like peptide 1 (GLP-1). Mol Metabol.

[5] Cornell S. (Sep 2020). A review of GLP-1 receptor agonists in type 2 diabetes: a focus on the mechanism of action of once-weekly agents. J Clin Pharm Therapeut.

[6] Almandoz J.P., Wadden T.A., Tewksbury C. (Jun 10 2024). Nutritional considerations with antiobesity medications. Obesity.

[7] Zhang X., Zhao Y., Chen S., Shao H. (Dec 2021). Anti-diabetic drugs and sarcopenia: emerging links, mechanistic insights, and clinical implications. J Cachexia Sarcopenia Muscle.

[8] Bettge K., Kahle M., Abd El Aziz M.S., Meier J.J., Nauck M.A. (Mar 2017). Occurrence of nausea, vomiting and diarrhoea reported as adverse events in clinical trials studying glucagon-like peptide-1 receptor agonists: a systematic analysis of published clinical trials. Diabetes Obes Metabol.

[9] Gorgojo-Martinez J.J., Mezquita-Raya P., Carretero-Gomez J. (Dec 24 2022). Clinical recommendations to manage gastrointestinal adverse events in patients treated with glp-1 receptor agonists: a multidisciplinary expert consensus. J Clin Med.

[10] Graneheim U.H., Lundman B. (Feb 2004). Qualitative content analysis in nursing research: concepts, procedures and measures to achieve trustworthiness. Nurse Educ Today.

[11] Elo S., Kyngas H. (Apr 2008). The qualitative content analysis process. J Adv Nurs.

[12] Ardavani A., Aziz H., Smith K., Atherton P.J., Phillips B.E., Idris I. (Jan 2021). The effects of very low energy diets and low energy diets with exercise training on skeletal muscle mass: a narrative review. Adv Ther.

[13] Driggin E., Goyal P. (Apr 2024). Malnutrition and sarcopenia as reasons for caution with GLP-1 receptor agonist use in HFpEF. J Card Fail.

[14] Chao A.M., Wadden T.A., Walsh O.A. (Dec 2019). Effects of liraglutide and behavioral weight loss on food cravings, eating behaviors, and eating disorder psychopathology. Obesity.

[15] Wadden T.A., Butryn M.L., Wilson C. (May 2007). Lifestyle modification for the management of obesity. Gastroenterology.

[16] Wadden T.A., Bailey T.S., Billings L.K. (Apr 13 2021). Effect of subcutaneous semaglutide vs placebo as an adjunct to intensive behavioral therapy on body weight in adults with overweight or obesity: the STEP 3 randomized clinical trial. JAMA.

[17] Jensen S.B.K., Blond M.B., Sandsdal R.M. (Mar 2024). Healthy weight loss maintenance with exercise, GLP-1 receptor agonist, or both combined followed by one year without treatment: a post-treatment analysis of a randomised placebo-controlled trial. EClinicalMedicine.

[18] Lundgren J.R., Janus C., Jensen S.B.K. (May 6 2021). Healthy weight loss maintenance with exercise, liraglutide, or both combined. N Engl J Med.

[19] Panza G.A., Armstrong L.E., Taylor B.A., Puhl R.M., Livingston J., Pescatello L.S. (Nov 2018). Weight bias among exercise and nutrition professionals: a systematic review. Obes Rev.

[20] Brewis A., SturtzSreetharan C., Wutich A. (Feb 13 2018). Obesity stigma as a globalizing health challenge. Glob Health.

[21] Tomiyama A.J., Carr D., Granberg E.M. (Aug 15 2018). How and why weight stigma drives the obesity 'epidemic' and harms health. BMC Med.

[22] Puhl R.M., Heuer C.A. (Jun 2010). Obesity stigma: important considerations for public health. Am J Publ Health.

[23] Mozaffarian D. (Mar 26 2024). GLP-1 agonists for obesity-A new recipe for success?. JAMA.

[24] Jones M., Eggett D., Bellini S.G., Williams P., Patten E.V. (Nov 2021). Patient-centered care: dietitians' perspectives and experiences. Patient Educ Counsel.

[25] Jortberg B.T., Fleming M.O. (Dec 2014). Registered dietitian nutritionists bring value to emerging health care delivery models. J Acad Nutr Diet.

[26] Sladdin I., Ball L., Bull C., Chaboyer W. (Aug 2017). Patient-centred care to improve dietetic practice: an integrative review. J Hum Nutr Diet.

[27] McKay N.J., Kanoski S.E., Hayes M.R., Daniels D. (Dec 2011). Glucagon-like peptide-1 receptor agonists suppress water intake independent of effects on food intake. Am J Physiol Regul Integr Comp Physiol.

